# Dose Distributions of an ^192^Ir Brachytherapy Source in Different Media

**DOI:** 10.1155/2014/946213

**Published:** 2014-04-07

**Authors:** C. H. Wu, Y. J. Liao, Y. W. Hsueh Liu, S. K. Hung, M. S. Lee, S. M. Hsu

**Affiliations:** ^1^Institute of Nuclear Engineering and Science, National Tsing Hua University, Hsinchu 300, Taiwan; ^2^School of Medical Laboratory Science and Biotechnology, College of Medical Science and Technology, Taipei Medical University, Taipei 110, Taiwan; ^3^School of Medicine, Tzu Chi University, Hualian 970, Taiwan; ^4^Department of Radiation Oncology, Buddhist Dalin Tzu Chi General Hospital, Chiayi 622, Taiwan; ^5^Department of Biomedical Imaging and Radiological Sciences, National Yang-Ming University, Taipei 112, Taiwan; ^6^Biophotonics and Molecular Imaging Research Center, National Yang-Ming University, Taipei 112, Taiwan

## Abstract

This study used MCNPX code to investigate the brachytherapy ^192^Ir dose distributions in water, bone, and lung tissue and performed radiophotoluminescent glass dosimeter measurements to verify the obtained MCNPX results. The results showed that the dose-rate constant, radial dose function, and anisotropy function in water were highly consistent with data in the literature. However, the lung dose near the source would be overestimated by up to 12%, if the lung tissue is assumed to be water, and, hence, if a tumor is located in the lung, the tumor dose will be overestimated, if the material density is not taken into consideration. In contrast, the lung dose far from the source would be underestimated by up to 30%. Radial dose functions were found to depend not only on the phantom size but also on the material density. The phantom size affects the radial dose function in bone more than those in the other tissues. On the other hand, the anisotropy function in lung tissue was not dependent on the radial distance. Our simulation results could represent valid clinical reference data and be used to improve the accuracy of the doses delivered during brachytherapy applied to patients with lung cancer.

## 1. Introduction

High dose rate (HDR) brachytherapy uses sealed radioactive sources to deliver radiation dose to a tumor over a short distance via intracavitary or interstitial placement. This methodology is designed to maximize the tumor dose while minimizing the dose to surrounding normal tissues. HDR brachytherapy is associated with a high dose gradient, with the dose decreasing rapidly away from the source. Several recent researches have studied the ^192^Ir source referred to in the American Association of Physics in Medicine Task Group 43 (AAPM TG-43) report [1]. That report on dose parameter calculations was based on the assumption of a water environment. Several researchers used a dosimeter and Monte Carlo (MC) code to investigate the dose distribution around the source [2–5].

The development of smaller radiation sources has widened the application of brachytherapy to cancers of the nasopharynx, esophagus, bronchus, lung, and esophagus [6–8]. Guilcher et al. pointed out that brachytherapy is an effective and safe treatment option for patients with endobronchial carcinoma who cannot receive surgery or external beam radiotherapy (EBRT) [9]. Koutcher et al. indicated that patients with locally recurrent nasopharynx cancer who received EBRT combined with brachytherapy had fewer severe late side effects compared with those treated with EBRT alone [10]. Brachytherapy has demonstrated good clinical efficacy, but dose calculations still assume that the treatment environment comprises a water medium around the source.

The AAPM TG-43 report provided dose calculation formulas and dose parameters for brachytherapy. Although that report can be used to evaluate the radiation dose received by soft tissues, human organs such as the nasopharynx, esophagus, bronchi, lungs, and bones have markedly differing densities. The AAPM TG-43 report does not provide the relevant dose parameters for these tissues, and, hence, the dose cannot be assessed accurately. This situation could result in tumor recurrence (due to an insufficient dose) or severe side effects in normal tissues (due to an excessive dose). Many of the dose parameters in the AAPM TG-43 report were calculated using MC calculations based on a water environment. The ^192^Ir dose distributions in bone and lung tissue are still unknown.

The main purpose of this study was to use the Monte Carlo N-Particle eXtended (MCNPX) code to calculate the dose distributions of an ^192^Ir source in water, bone, and lung tissue and to perform radiophotoluminescent glass dosimeter measurements in tissue phantoms. The measured values were compared with the results obtained using MCNPX code to examine the dose distributions in different tissues. The results indicate that the dose parameters calculated by MCNPX code can be used as a reference for clinical brachytherapy.

## 2. Materials and Methods

### 2.1. ^192^Ir Source

**Figure 1:**
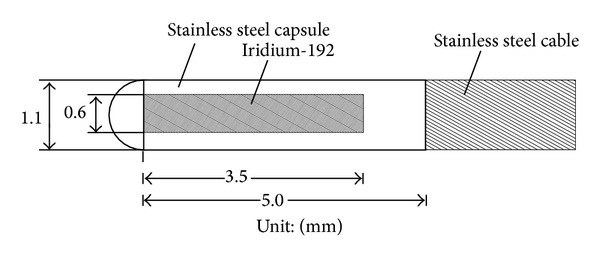
Structural diagram of the Nucletron ^192^Ir microSelectron HDR source and its stainless steel outer cover.

The photon energy spectrum of ^192^Ir is quite complex, containing energies ranging from 0.0089 to 1.0615 MeV. ^192^Ir has a relatively high atomic number (*Z* = 77) and density (*ρ* = 22.42 g/cm^3^), and the dose distribution around the source depends on its dimensions and outer encapsulation, as well as the treatment environment. The ^192^Ir source used in this study had an active length of 3.5 mm and a diameter of 0.6 mm, as shown in Figure 1. It was encapsulated by a stainless steel outer cover with an outer diameter of 1.1 mm that was welded to a steel cable for attachment to a remote after-loading machine (microSelectron HDR, Nucletron, The Netherlands). For the purpose of dose calculation, the stainless steel cable extended 2 mm from the outer cover on the proximal side of the active source.

### 2.2. Monte-Carlo Simulation

This study used the MCNPX 2.70 code to calculate the dose distributions of an ^192^Ir source in water, bone, and lung tissue. The photon energy spectrum of ^192^Ir was obtained from Brookhaven National Laboratory [13]. The simulation was divided into three parts. First, a 30 cm diameter spherical phantom was used in simulations for the radial dose function and anisotropy function; the spherical dimensions used in the simulations were identical to those used by Williamson and Li [11] and Karaiskos et al. [12], so that our results could be compared to the results calculated by those authors. The F6 tally was used to speed up the calculation, and the source was set at the center of the phantom. The F6 tally based on the assumption of an electronic balance exists in the tally region. The electron will lose its energy instead of undergoing electron transport at the position where a photon and electron collide. The radiation particles were removed from the simulation when they moved outside of the phantom. At least 10^8^ particles were simulated, yielding 1*σ* statistic errors of less than 3% for the total dose. Second, the phantom was simulated as a cylinder with a diameter of 25 cm and a height of 25 cm in order to closely approximate the experimental phantom, and the absorbed doses were recorded in a two-dimensional (2D) matrix. Third, the influence of phantom size on the radial dose function was investigated by simulating spherical water, lung, and bone phantoms with various radii.

### 2.3. Phantoms

The compositions of bone and lung tissue used in this study are based on the ICRU 44 (International Commission on Radiation Units and Measurements Report 44) recommendation [14]. Cortical bone and inflated lung were adopted as bone and lung phantoms with densities of 1.92 and 0.26 g/cm^3^, respectively, while a polystyrene phantom with a density of 1.04 g/cm^3^ was used to represent water.

### 2.4. Radiophotoluminescent Glass Dosimeter Measurements

The radiophotoluminescent glass dosimeters used in this study (GD-301, Asahi Techno Glass Corporation, Shizuoka, Japan) had the following weight composition: 31.55% P, 51.16% O, 6.12% Al, 11.00% Na, and 0.17% Ag. The effective atomic number and physical density of the dosimeters were 12.04 and 2.61 g/cm^3^, respectively [15]. The GD-301 dosimeter is composed of a rod glass element measuring 1.5 mm in diameter and 8.5 mm in length, and it must undergo heat treatment at 70°C for 1 hour before reading with the Dose Ace FGD-100 reader. The active readout size of the GD-301 dosimeter of 1 mm makes it a suitable tool for measuring brachytherapy sources with high dose gradients [16].

**Figure 2:**
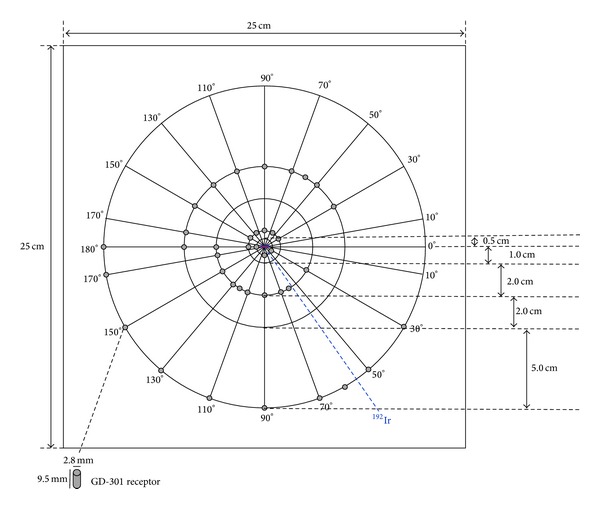
Locations for GD-301 radiophotoluminescent glass dosimeter measurements.

To support the MC simulation results, the radial dose function and anisotropy function were measured using radiophotoluminescent glass dosimeters in phantoms representing three different tissue types. Slabs were sandwiched together to build 25 cm × 25 cm × 25 cm phantoms, in which a slot was milled in the center to accommodate the ^192^Ir source. For GD-301 dosimeter measurements, concentric holes were drilled along polar angles of *θ* = 0–180° at radial distances of *r* = 0.5, 1.0, 3.0, 5.0, and 10.0 cm from the center of source, as shown in Figure 2.

## 3. Results and Discussion

### 3.1. Radial Dose Function and Dose-Rate Constant

**Figure 3:**
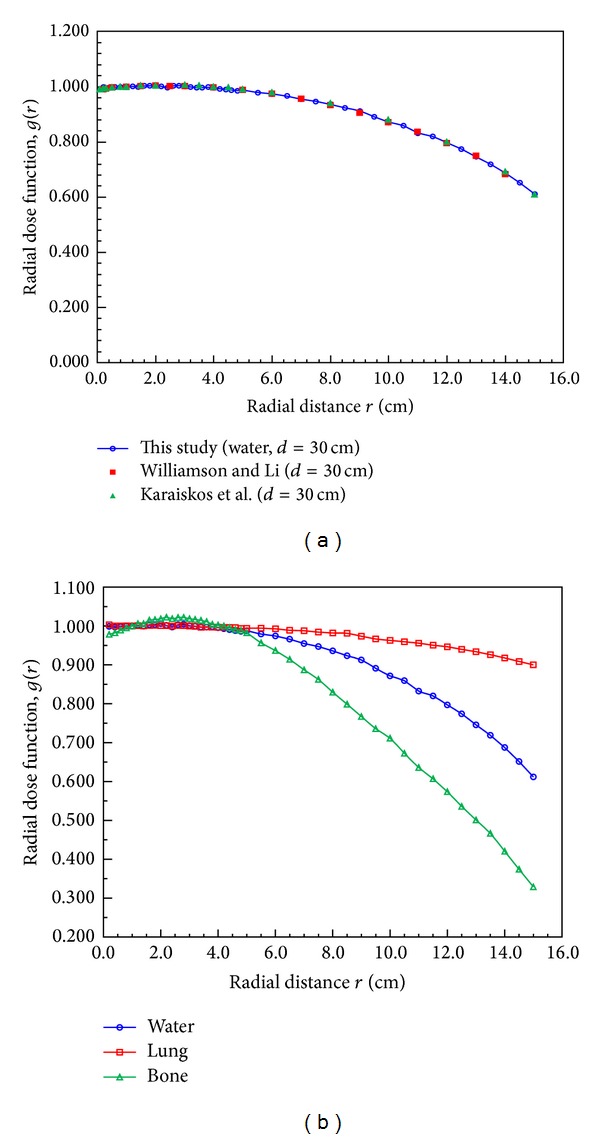
Radial dose function calculated by MCNPX code with the ^192^Ir microSelectron HDR source centered in three spherical tissue phantoms with a diameter of 30 cm: (a) comparison of MCNPX results and previously reported results and (b) radial dose functions in water, bone, and lung tissue.

The radial dose function, *g*(*r*), accounts for the effects of photon absorption and scatter in the medium along the transverse axis of the source. MCNPX results of *g*(*r*) for a 30 cm diameter spherical phantom are presented in Figure 3(a) and Table 1; these results are highly consistent with the MC results of Williamson and Li and Karaiskos et al. (within 2%). The dose-rate constant, Λ, calculated in water was 1.115 ± 0.010 cGy h^−1^ U^−1^ (1 U = 1 cGy cm^2^ h^−1^), also highly consistent with the MC results of Williamson and Li and Karaiskos et al., as presented in Table 2.

**Figure 4:**
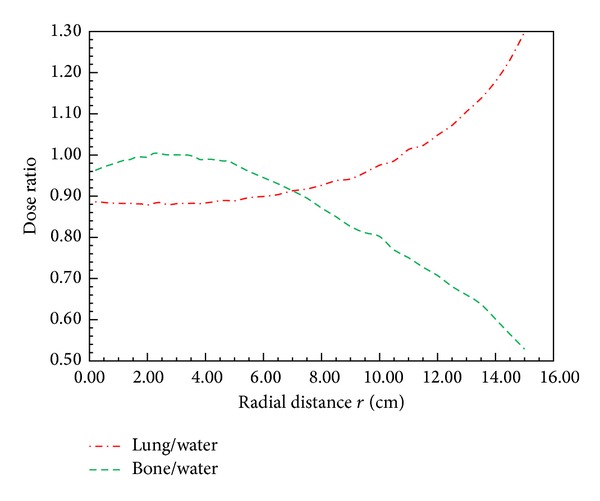
Radial dose ratio calculated by MCNPX code with the ^192^Ir microSelectron HDR source centered in three spherical tissue phantoms with a diameter of 30 cm. The red line represents the dose ratio of lung to water; the green line represents the dose ratio of bone to water.

Figure 3(b) presents the radial dose functions calculated for water, bone, and lung phantoms. As the depth increased, the radial dose function decreased more slowly for lung than for water due to the linear attenuation coefficient being small in lung tissue, whereas the function decreased faster in bone than in water due to the linear attenuation efficient being higher in bone than in water. Tables 1 and 2 also present the radial dose functions and dose-rate constants of bone and lung tissue to provide clinical reference data for dose calculations in various tissue types around an ^192^Ir source. The ratios of the dose calculated by MCNPX code in lung tissue and bone to the dose in water are shown in Figure 4. Up to a depth of 11 cm the dose was less in lung tissue than in water; for example, at a depth of 2.8 cm, the lung/water dose ratio was 0.88. This shows that a treatment planning system (TPS) would overestimate the lung dose by 12%. Moreover, at a depth of 15 cm, the lung dose would be underestimated by 30%. These results also imply that, if a tumor is located in the lung, the tumor dose will be overestimated if the different tissue densities are not taken into account. At depths of less than 5 cm the dose rate in bone was similar to that in water, whereas at depths greater than 5 cm the bone dose would be overestimated by 47%.

### 3.2. Influence of Phantom Dimensions on the Radial Dose Function

**Figure 5:**
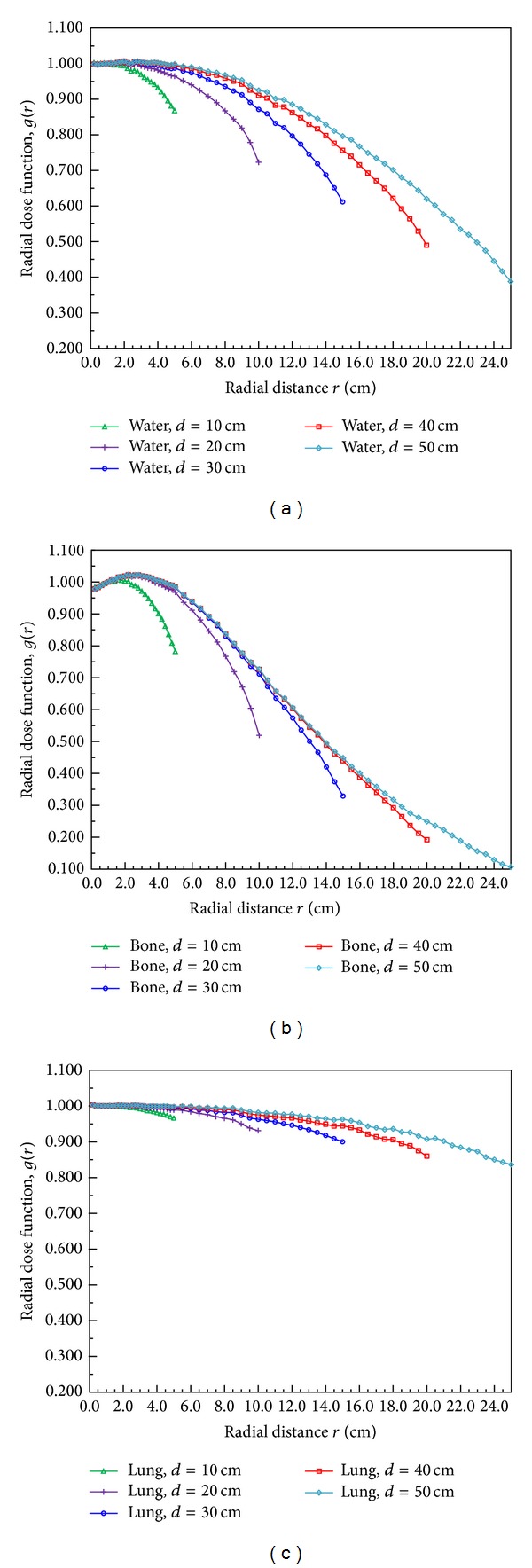
Radial dose functions calculated for spherical phantoms of the three tissue types with different diameters (*d* = 10–50 cm): (a) water, (b) bone, and (c) lung.

Karaiskos et al. used MC calculations for an ^192^Ir microSelectron source to examine the dose parameters in spherical water phantoms with different diameters. They found that the phantom dimensions significantly affected the radial dose functions near to the edges of the phantom, with deviations of up to 25% being observed. They did not observe that the anisotropy functions depended significantly on the phantom size. The present study simulated spherical water, bone, and lung phantoms with diameters ranging from 10 to 50 cm in order to evaluate how the phantom dimensions influence the radial dose functions in these tissues.  Figure 5 shows the radial dose functions in water near the edge of the phantom, where deviations of up to 23% are evident and the results are similar to those of Karaiskos et al. The deviations in the radial dose functions in bone and lung tissue were 26.7% and 6.5%, respectively. These results show that the backscattered components vary with the material densities, being maximal in bone and minimal in lung tissue. Moreover, the backscattering in lung tissue does not differ significantly with the phantom size.

### 3.3. Anisotropy Function Comparison

**Figure 6:**
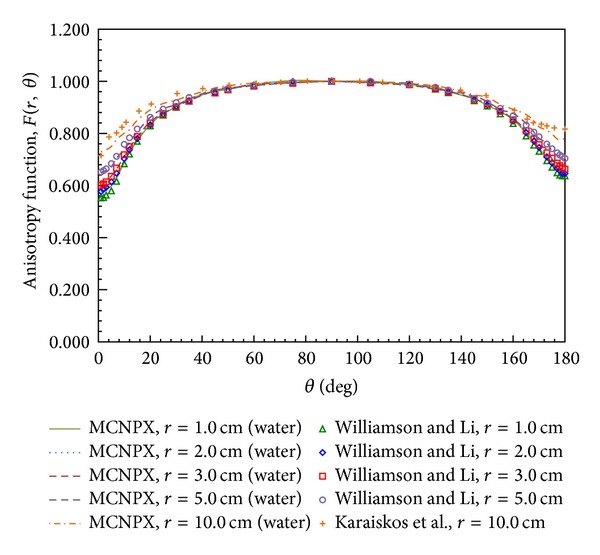
Anisotropy functions calculated by MCNPX code at *r* = 1, 2, 3, 5 and 10 cm from the source center. The MC results of Williamson and Li and Karaiskos et al. are also plotted for comparison.

The anisotropy function, *F*(*r*, *θ*), accounts for the anisotropy of the dose distribution around the source due to the geometry structure of the source and the encapsulation. Our MCNPX results of *F*(*r*, *θ*) for a 30 cm diameter spherical phantom are presented in Figure 6 and Table 3. The calculated results were compared with the MC calculations of Williamson and Li; the differences were within 4.6% for *r* = 1 cm and *θ* < 5°, within 2.5% for *r* = 1 cm and 5° < *θ* < 180°, and within 2% for *r* > 1 cm. Moreover, our results agree with the MC calculations of Karaiskos et al. within 2.7% for 15.7° < *θ* < 178° and within 5% for *θ* < 5° and *θ* = 179°. These comparisons indicate that our calculation results are highly consistent with the MC results of Williamson and Li and Karaiskos et al.

**Figure 7:**
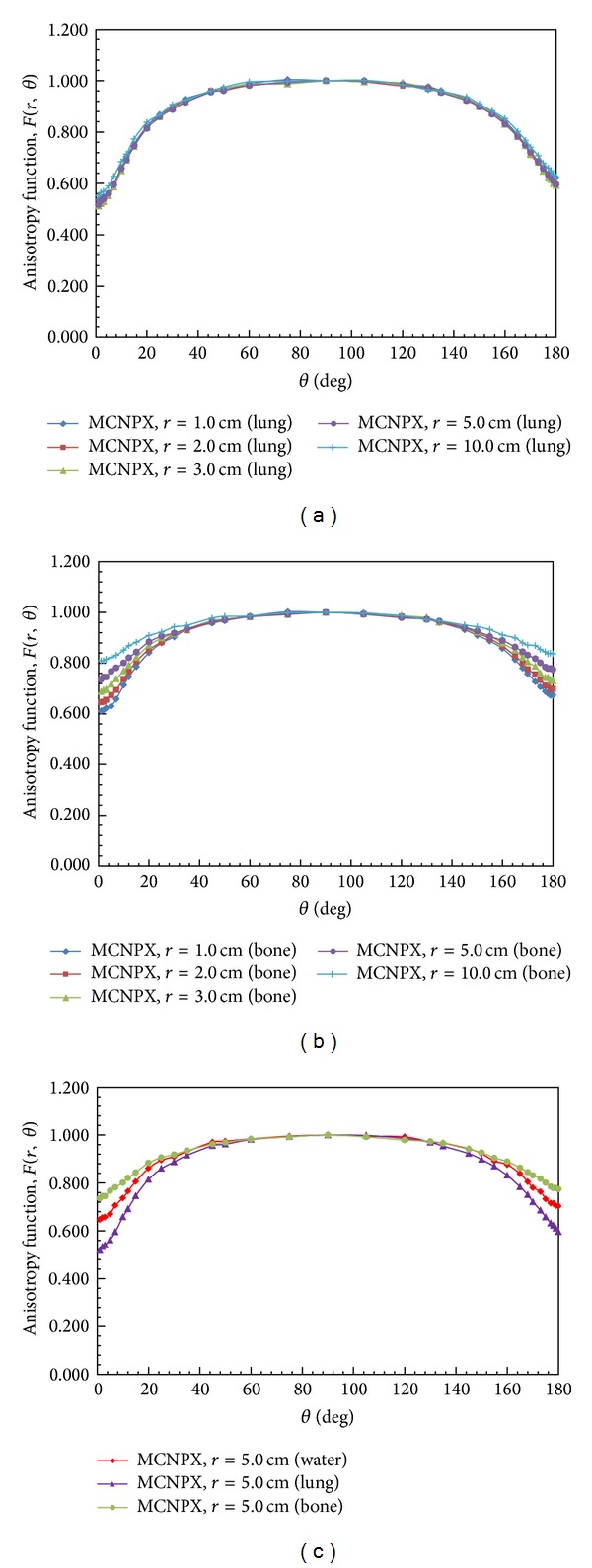
Comparison of anisotropy functions for phantoms of three tissue types: (a) lung at *r* = 1, 2, 3, 5 and 10 cm; (b) bone at *r* = 1, 2, 3, 5 and 10 cm; and (c) water, bone, and lung tissue at *r* = 5 cm.

The anisotropy functions in the three tissue types are shown in Figure 7.  Figure 7(a) plots our calculation results for ^192^Ir in lung tissue as an anisotropy function, with the data showing that *F*(*r*, *θ*) is independent of the radial distance. However, *F*(*r*, *θ*) was closer to 1 in bone tissue for all polar angles when radial distance increases, as shown in Figure 7(b).  Figure 7(c) presents our anisotropy functions in the three tissue types at *r* = 5 cm for comparison. The anisotropy function increases due to the increasing contribution of scattered component in medium which compensate for the attenuation of primary radiation. *F*(*r*, *θ*) in bone resembles a point source due to the scattered components being larger than those in water and lung tissue. This study used MCNPX code to calculate the dose parameters in bone and lung tissue for clinical reference; the anisotropy functions in bone and lung tissue are listed in Table 3.

### 3.4. Radiophotoluminescent Glass Dosimeter Measurements and 2D Dose Distribution

**Figure 8:**
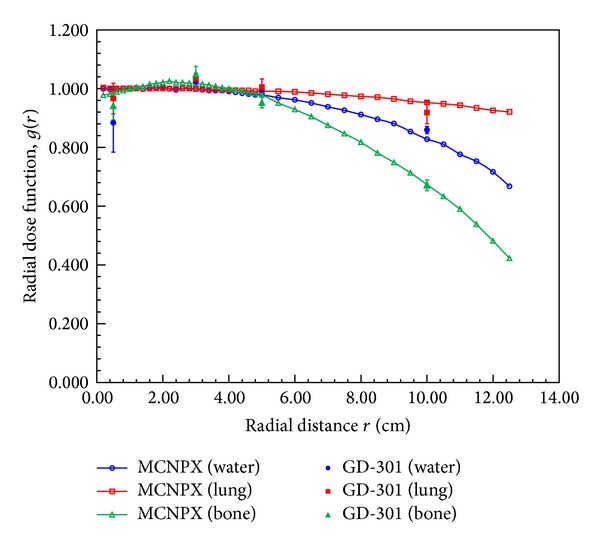
Averaged values of GD-301 dosimeter radial dose function measurements compared with the MCNPX results for phantoms of three tissue types (water, bone, and lung tissue).

**Figure 9:**
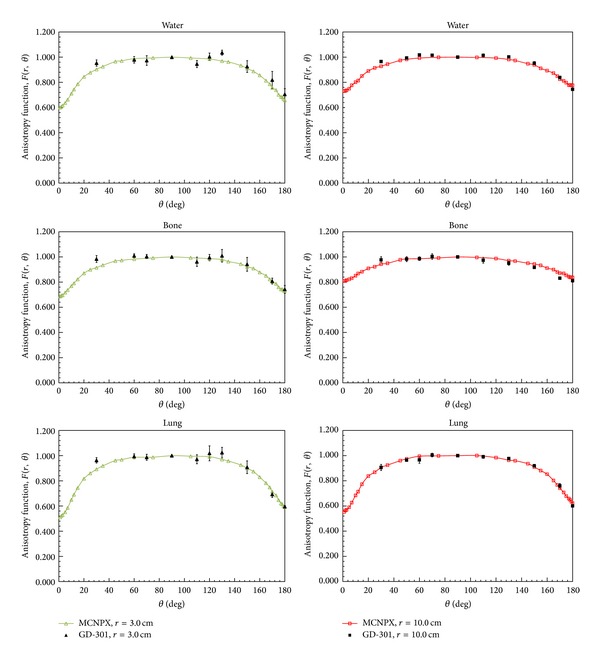
Averaged values of GD-301 dosimeter anisotropy function measurements compared with the MCNPX results for phantoms of three tissue types (water, bone, and lung tissue) at radial distances of *r* = 3 and 10 cm.

To our knowledge there are no previous reports of the dose parameters for bone and lung tissue. We used the MCNPX code to perform dose calculations for three different types of tissue and we also used the GD-301 dosimeter to measure the dose in order to verify the reliability of the obtained MCNPX results. Our measured data are averaged values from three measurements at each radial distance. Comparisons of the measured values and the MCNPX results are presented in Figures 8 and 9. For the radial dose function, the maximum difference between the measured value and the MCNPX results for water was 11.4% at *r* = 0.5 cm. This difference was due to the high dose gradient of ^192^Ir. The statistical error of the measured value decreases as the radial distance increases. The statistical error for all MC calculations was within 3% (1*σ*).

**Figure 10:**
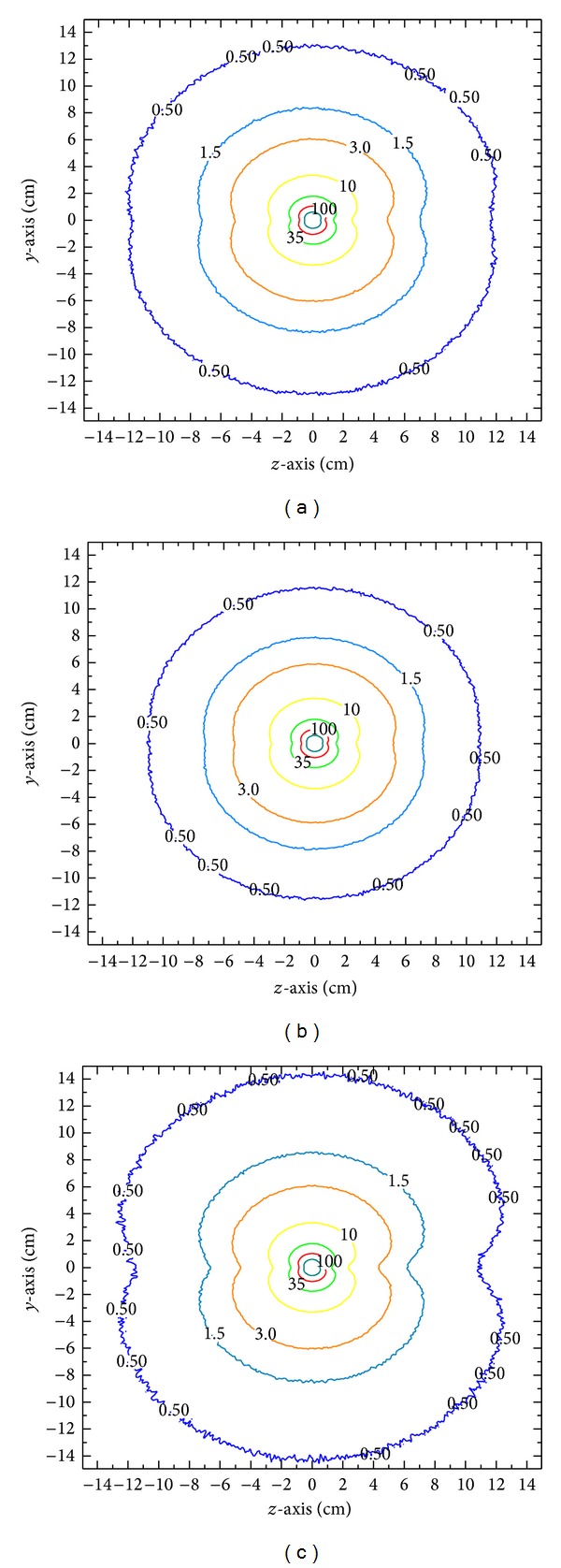
2D isodose curves for (a) water, (b) bone, and (c) lung.

This study also used MCNPX code to calculate the 2D dose distribution for the three tissue types, as shown in Figure 10. The dose was normalized to that in the water phantom at *r* = 1 cm. Figures 10(a)–10(c) present the relative dose distributions in water, bone, and lung phantoms. The results reveal that the dose distribution in bone remains close to that of a point source as the radial distance increases and that the rate of dose attenuation was the fastest among three different types of tissue (due to the linear attenuation coefficient).

## 4. Conclusions

This study used MCNPX code to calculate the ^192^Ir dose distributions in water, bone, and lung tissue. The dose parameters for water were highly consistent with the MC results of Williamson and Li and Karaiskos et al. For *r* > 3 cm, the results from the MCNPX code and GD-301 dosimeter measurements for bone and lung tissue were highly consistent. The results demonstrate that the dose distribution of HDR brachytherapy differed in water, bone, and lung tissue. The TPS, which currently does not take into account such differences in tissue density, thus, overestimated the dose by up to 12% in lung tissue near the source. Moreover, the magnitude of the attenuation and scatter would vary with the tissue density. The radial dose functions would depend not only on the phantom size but also on the phantom density. The dose-rate constant, radial dose function, and anisotropy function have been calculated for the ^192^Ir microSelectron source in water, bone, and lung tissue. These dose parameters can be used as clinical reference data and to improve the accuracy of the doses delivered during HDR brachytherapy.

## Figures and Tables

**Table 1:** Radial dose function, *g*(*r*), calculated by MCNPX code for the ^192^Ir microSelectron source centered in a spherical tissue phantom with *d* = 30 cm.

Radial distance (cm)	Water	Bone	Lung	Radial distance (cm)	Water	Bone	Lung
0.2	0.999	0.978	1.003	4.8	0.986	0.989	0.994
0.4	0.997	0.982	1.000	5.0	0.988	0.983	0.994
0.6	0.998	0.989	1.000	5.5	0.979	0.956	0.994
0.8	1.000	0.995	1.000	6.0	0.974	0.937	0.993
1.0	1.000	1.000	1.000	6.5	0.966	0.914	0.989
1.2	1.001	1.006	1.001	7.0	0.955	0.887	0.988
1.4	1.000	1.006	1.000	7.5	0.947	0.863	0.984
1.6	1.003	1.015	1.001	8.0	0.936	0.830	0.982
1.8	1.004	1.016	1.002	8.5	0.923	0.799	0.981
2.0	1.006	1.018	1.001	9.0	0.913	0.767	0.974
2.2	1.001	1.023	1.001	9.5	0.891	0.736	0.966
2.4	0.997	1.019	1.000	10.0	0.872	0.711	0.963
2.6	1.003	1.022	1.001	10.5	0.859	0.672	0.959
2.8	1.004	1.022	1.001	11.0	0.833	0.636	0.956
3.0	1.001	1.019	1.000	11.5	0.820	0.607	0.950
3.2	0.999	1.017	0.999	12.0	0.797	0.574	0.947
3.4	0.997	1.015	0.997	12.5	0.774	0.536	0.940
3.6	0.997	1.011	0.997	13.0	0.746	0.501	0.934
3.8	0.998	1.004	0.997	13.5	0.719	0.467	0.926
4.0	0.996	1.002	0.997	14.0	0.687	0.421	0.918
4.2	0.993	1.000	0.996	14.5	0.651	0.374	0.909
4.4	0.991	0.995	0.996	15.0	0.612	0.329	0.900
4.6	0.988	0.991	0.996				

**Table 2:** Dose-rate constant, Λ, calculated by MCNPX code for the ^192^Ir microSelectron source centered in a spherical tissue phantom with *d* = 30 cm.

Author	Medium	Λ (cGy h^−1^ U^−1^)
Williamson and Li [11]	30 cm water phantom	1.115 (±0.5%)
Karaiskos et al. [12]	30 cm water phantom	1.116 (±0.5%)

This study	30 cm water phantom	1.115 (±0.9%)
	30 cm bone phantom	1.097 (±0.9%)
	30 cm lung phantom	0.984 (±0.9%)

**Table 3:** Anisotropy function, *F*(*r*, *θ*), calculated by MCNPX code for the ^192^Ir microSelectron source centered in a spherical tissue phantom with *d* = 30 cm.

Polar angle (degrees)	Water						Lung						Bone					
	Radial distance from active source center						Radial distance from active source center						Radial distance from active source center					
	0.5 cm	1.0 cm	2.0 cm	3.0 cm	5.0 cm	10.0 cm	0.5 cm	1.0 cm	2.0 cm	3.0 cm	5.0 cm	10.0 cm	0.5 cm	1.0 cm	2.0 cm	3.0 cm	5.0 cm	10.0 cm
1	0.629	0.577	0.579	0.601	0.648	0.730	0.597	0.536	0.514	0.512	0.517	0.553	0.635	0.612	0.646	0.687	0.736	0.809
2	0.631	0.579	0.583	0.607	0.655	0.733	0.600	0.539	0.519	0.523	0.534	0.564	0.637	0.614	0.648	0.694	0.745	0.809
3	0.635	0.589	0.591	0.616	0.657	0.737	0.603	0.549	0.527	0.529	0.540	0.569	0.640	0.623	0.656	0.697	0.745	0.815
5	0.647	0.598	0.611	0.637	0.671	0.750	0.617	0.558	0.549	0.551	0.562	0.590	0.653	0.630	0.674	0.717	0.767	0.822
7	0.666	0.628	0.638	0.663	0.706	0.776	0.638	0.593	0.583	0.586	0.595	0.627	0.671	0.658	0.694	0.738	0.781	0.831
10	0.722	0.688	0.698	0.711	0.737	0.801	0.699	0.660	0.650	0.650	0.658	0.684	0.722	0.713	0.738	0.769	0.801	0.851
12	0.750	0.726	0.729	0.744	0.766	0.814	0.730	0.703	0.691	0.690	0.691	0.714	0.749	0.746	0.766	0.791	0.821	0.868
15	0.793	0.771	0.778	0.786	0.806	0.850	0.775	0.753	0.746	0.744	0.746	0.773	0.790	0.786	0.807	0.823	0.844	0.882
20	0.846	0.835	0.838	0.845	0.861	0.890	0.833	0.823	0.818	0.818	0.814	0.837	0.841	0.841	0.854	0.870	0.884	0.909
25	0.882	0.875	0.873	0.877	0.896	0.915	0.871	0.868	0.856	0.860	0.860	0.868	0.875	0.880	0.882	0.899	0.906	0.922
30	0.912	0.901	0.907	0.904	0.909	0.927	0.903	0.896	0.899	0.892	0.888	0.906	0.905	0.903	0.913	0.915	0.918	0.943
35	0.932	0.933	0.927	0.925	0.932	0.945	0.925	0.929	0.919	0.919	0.915	0.925	0.926	0.932	0.929	0.935	0.935	0.949
45	0.961	0.961	0.965	0.965	0.970	0.974	0.955	0.958	0.962	0.961	0.955	0.960	0.955	0.958	0.962	0.968	0.962	0.977
50	0.971	0.968	0.972	0.971	0.974	0.983	0.968	0.969	0.968	0.968	0.961	0.974	0.967	0.967	0.974	0.973	0.969	0.984
60	0.987	0.988	0.983	0.988	0.984	0.993	0.985	0.988	0.981	0.987	0.981	0.995	0.985	0.984	0.982	0.983	0.983	0.986
75	0.996	1.003	0.994	0.994	0.995	0.999	0.996	1.004	0.994	0.987	0.993	0.998	0.996	1.003	0.995	0.992	0.992	0.992
90	1.000	1.000	1.000	1.000	1.000	1.000	1.000	1.000	1.000	1.000	1.000	1.000	1.000	1.000	1.000	1.000	1.000	1.000
105	0.997	1.000	0.997	0.993	0.995	0.998	0.997	1.001	0.995	0.995	0.998	1.001	0.997	0.998	0.993	0.993	0.992	0.996
120	0.985	0.987	0.983	0.985	0.992	0.993	0.983	0.987	0.979	0.991	0.986	0.984	0.984	0.985	0.984	0.986	0.979	0.987
130	0.970	0.978	0.975	0.969	0.970	0.982	0.967	0.976	0.973	0.970	0.969	0.964	0.966	0.975	0.973	0.979	0.973	0.970
135	0.961	0.961	0.959	0.963	0.966	0.973	0.956	0.961	0.955	0.957	0.954	0.959	0.956	0.961	0.960	0.964	0.966	0.967
145	0.931	0.932	0.936	0.933	0.942	0.948	0.924	0.930	0.928	0.926	0.922	0.936	0.925	0.931	0.938	0.944	0.940	0.950
150	0.913	0.908	0.911	0.910	0.922	0.939	0.905	0.901	0.901	0.899	0.898	0.909	0.907	0.909	0.918	0.921	0.927	0.943
155	0.887	0.884	0.887	0.888	0.891	0.911	0.876	0.876	0.874	0.875	0.870	0.881	0.881	0.887	0.898	0.909	0.905	0.933
160	0.853	0.853	0.854	0.856	0.877	0.893	0.840	0.842	0.834	0.830	0.832	0.852	0.847	0.858	0.868	0.877	0.890	0.911
165	0.813	0.803	0.805	0.814	0.839	0.873	0.798	0.788	0.780	0.782	0.783	0.803	0.809	0.813	0.828	0.851	0.864	0.900
168	0.784	0.767	0.775	0.786	0.805	0.849	0.767	0.748	0.745	0.748	0.750	0.767	0.781	0.780	0.801	0.823	0.845	0.880
170	0.763	0.743	0.746	0.757	0.780	0.825	0.745	0.723	0.710	0.714	0.721	0.741	0.762	0.758	0.776	0.804	0.832	0.871
173	0.730	0.707	0.719	0.739	0.763	0.812	0.711	0.684	0.680	0.685	0.685	0.708	0.730	0.727	0.756	0.787	0.818	0.869
175	0.713	0.686	0.694	0.702	0.733	0.797	0.693	0.662	0.651	0.647	0.658	0.678	0.714	0.706	0.733	0.760	0.801	0.854
177	0.701	0.663	0.668	0.685	0.716	0.779	0.679	0.637	0.622	0.621	0.632	0.659	0.700	0.688	0.712	0.743	0.784	0.844
178	0.698	0.656	0.663	0.681	0.715	0.776	0.676	0.630	0.618	0.615	0.622	0.649	0.698	0.681	0.711	0.743	0.778	0.837
179	0.695	0.647	0.648	0.667	0.705	0.780	0.673	0.622	0.599	0.598	0.610	0.635	0.695	0.673	0.697	0.733	0.779	0.839
180	0.692	0.649	0.646	0.658	0.703	0.777	0.671	0.622	0.595	0.592	0.596	0.623	0.693	0.674	0.699	0.730	0.774	0.837
